# The effect of recombination on the evolution of a population of *Neisseria meningitidis*

**DOI:** 10.1101/gr.264465.120

**Published:** 2021-07

**Authors:** Neil MacAlasdair, Maiju Pesonen, Ola Brynildsrud, Vegard Eldholm, Paul A. Kristiansen, Jukka Corander, Dominique A. Caugant, Stephen D. Bentley

**Affiliations:** 1 Parasites and Microbes, Wellcome Sanger Institute, Cambridge CB10 1SA, United Kingdom;; 2 Oslo Centre for Biostatistics and Epidemiology (OCBE), Oslo University Hospital Research Support Services, Blindern, 0317 Oslo, Norway;; 3 Division for Infection Control and Environmental Health, Norwegian Institute of Public Health, 0213 Oslo, Norway;; 4 Department of Food Safety and Infection Biology, Faculty of Veterinary Science, Norwegian University of Life Science, 0454 Oslo, Norway;; 5 University of Oslo, Department of Biostatistics, Blindern, 0317 Oslo, Norway;; 6 Helsinki Institute for Information Technology HIIT, Department of Mathematics and Statistics, University of Helsinki, 00014 Helsinki, Finland;; 7 Department of Community Medicine, Faculty of Medicine, University of Oslo, Blindern, 0316 Oslo, Norway

## Abstract

*Neisseria meningitidis* (the meningococcus) is a major human pathogen with a history of high invasive disease burden, particularly in sub-Saharan Africa. Our current understanding of the evolution of meningococcal genomes is limited by the rarity of large-scale genomic population studies and lack of in-depth investigation of the genomic events associated with routine pathogen transmission. Here, we fill this knowledge gap by a detailed analysis of 2839 meningococcal genomes obtained through a carriage study of over 50,000 samples collected systematically in Burkina Faso, West Africa, before, during, and after the serogroup A vaccine rollout, 2009–2012. Our findings indicate that the meningococcal genome is highly dynamic, with highly recombinant loci and frequent gene sharing across deeply separated lineages in a structured population. Furthermore, our findings illustrate how population structure can correlate with genome flexibility, as some lineages in Burkina Faso are orders of magnitude more recombinant than others. We also examine the effect of selection on the population, in particular how it is correlated with recombination. We find that recombination principally acts to prevent the accumulation of deleterious mutations, although we do also find an example of recombination acting to speed the adaptation of a gene. In general, we show the importance of recombination in the evolution of a geographically expansive population with deep population structure in a short timescale. This has important consequences for our ability to both foresee the outcomes of vaccination programs and, using surveillance data, predict when lineages of the meningococcus are likely to become a public health concern.

*Neisseria meningitidis*, the meningococcus, is a species of bacteria found exclusively in humans. It can cause meningitis, an infection of the membranes covering the brain and spinal cord, as well as septicemia (Stephens et al. 2007). These infections are difficult to treat, even with antimicrobials, and have a high case fatality rate. Of the 12 serogroups defined on the basis of the structure of the capsular polysaccharide, six (A, B, C, W, X, and Y) are responsible for nearly all cases of invasive meningococcal disease (IMD) worldwide. In contrast to strains that are capable of causing disease, non-disease-causing carriage isolates are typically unencapsulated. However, most infections of encapsulated and unencapsulated *N. meningitidis* are asymptomatic, with the bacteria being carried in the oropharynx of human populations without causing disease with a prevalence of ∼5%–10% (Christensen et al. 2010). It is likely that essentially all individuals will be colonized by potentially IMD-causing bacteria once or even several times during their lifetimes, so there are an uncertain number of carriage infections and transmission events in a human population. This presents a challenge for controlling the disease, and in order to reduce the incidence of IMD, effective polysaccharide-conjugate vaccines against serogroups A, C, W, and Y have been developed and introduced in national vaccination programs. These vaccines are, however, expensive and not affordable for low-income countries. Therefore, a monovalent conjugate serogroup A vaccine was produced and successfully introduced in large-scale vaccination campaigns in countries of the so-called “meningitis belt” of sub-Saharan Africa (Diomandé et al. 2015; Trotter et al. 2017) a region stretching from the Gambia and Senegal to Ethiopia (Molesworth et al. 2002).

Prior to the vaccination campaigns that started at the end of 2010, the overall incidence of meningococcal meningitis in the region was substantially higher than anywhere else in the world and included epidemics that occurred in the winter months every five to 12 years (Trotter and Greenwood 2007). Though the vaccine has been very effective at controlling meningitis epidemics caused by serogroup A, the main cause of IMD in the meningitis belt (Diomandé et al. 2015; Trotter et al. 2017), other serogroups (C, W, and X) have emerged or expanded in the region, reducing the initial impact of the vaccine (Topaz et al. 2019). There is also concern that virulent strains circulating in the population might switch capsule or that less virulent strains not covered by the current vaccine might acquire virulence genes (Bårnes et al. 2017; Brynildsrud et al. 2019).

Both of these potential scenarios are driven by the ability of bacteria from the genus *Neisseria* to be naturally transformable and to readily recombine their DNA with one another (Obergfell and Seifert 2015), in concert with selection (Arnold et al. 2020). Various mechanisms of recombination have been described in *N. meningitidis* (Schoen et al. 2009; Marri et al. 2010; Joseph et al. 2011), involving abundant and diverse repetitive DNA sequences in its chromosome. The evolutionary and epidemiological effects of recombination in *N. meningitidis* have been studied in some detail (Marri et al. 2010; Joseph et al. 2011; Retchless et al. 2018), but less work has been undertaken to describe how the extent of recombination varies both between different lineages of *N. meningitidis* and between different regions of its complete genome in a single circulating carriage population. In particular, there is little understanding of how this recombination affects the process of natural selection. Studies across diverse populations and species have suggested that there is likely some variation (Castillo-Ramírez et al. 2012; Ezewudo et al. 2015), and this variation in recombination rate is particularly relevant amid the disruption of population structure caused by large-scale vaccine introduction (Potts et al. 2018).

Burkina Faso, located in the center of the meningitis belt, historically has had a high burden of disease caused by serogroup A meningococci (Nicolas et al. 2005) and was one of the first countries to introduce the serogroup A conjugate vaccine in a mass vaccination campaign in 2010 (Kristiansen et al. 2013). Since then, the incidence of IMD has decreased overall, but there have been meningitis outbreaks caused by serogroups W and X, belonging, respectively, to the sequence types (STs) 11 and 181 (Kristiansen et al. 2013).

Here, we present a detailed population genetic analysis, focusing on recombination, in a collection of 2838 *N. meningitidis* carriage isolates collected from three areas of Burkina Faso over the course of the implementation of the serogroup A vaccine, from 2009 to 2012. This collection has been previously studied using molecular typing techniques (Kristiansen et al. 2013, 2014) which identified ST-181 as the dominant lineage in this population during the time period when sampling was performed. It further showed that the vaccine was effective at reducing the incidence of its target, serogroup A isolates, with none of the previously prevalent serogroup A ST-2859 clone detected after vaccination. This was, however, associated with an increase in the incidence of the disease-causing serogroup X ST-181 complex and also with the introduction and expansion of the disease-causing serogroup W ST-11complex. In this study, we use whole-genome sequencing with Illumina short-read technology to generate de novo assemblies for each isolate, which are then used to construct phylogenies, infer recombination events, and perform tests for selection. Our study recapitulates the finding of previous molecular studies on this population, but leveraging whole-genome data, this study additionally sought to determine whether lineages that make up this population have significantly different recombination rate phenotypes; if different lineages have specific recombination hotspot regions—loci where much more recombination takes place compared to elsewhere in their genomes; to what extent recombination is occurring between the different lineages which make up this population; and finally, whether it is possible to ascertain the evolutionary causes and effects for recombination in this population. Though recombination in *N. meningitidis* has been a known phenomenon and studied for some time (Zhu et al. 1999), we believe this study to be the first to detail the extent of variation in recombination rate within a sampled population and to characterize how and why recombination affects the evolution of the population.

## Results

### Population structure

**Figure 1.**
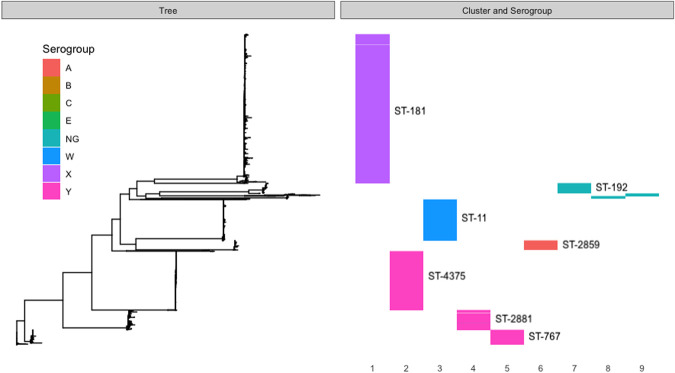
Core genome phylogeny, annotated with PopPUNK-inferred clusters, serogroups, and sequence types for all Burkina Faso isolates in the collection. Clusters are numbered 1–9 from *left* to *right*, serogroups are indicated with colors as per the legend, and sequence types for the seven largest clusters are indicated with labels. NG indicates that the isolates were nonserogroupable.

**Figure 2.**
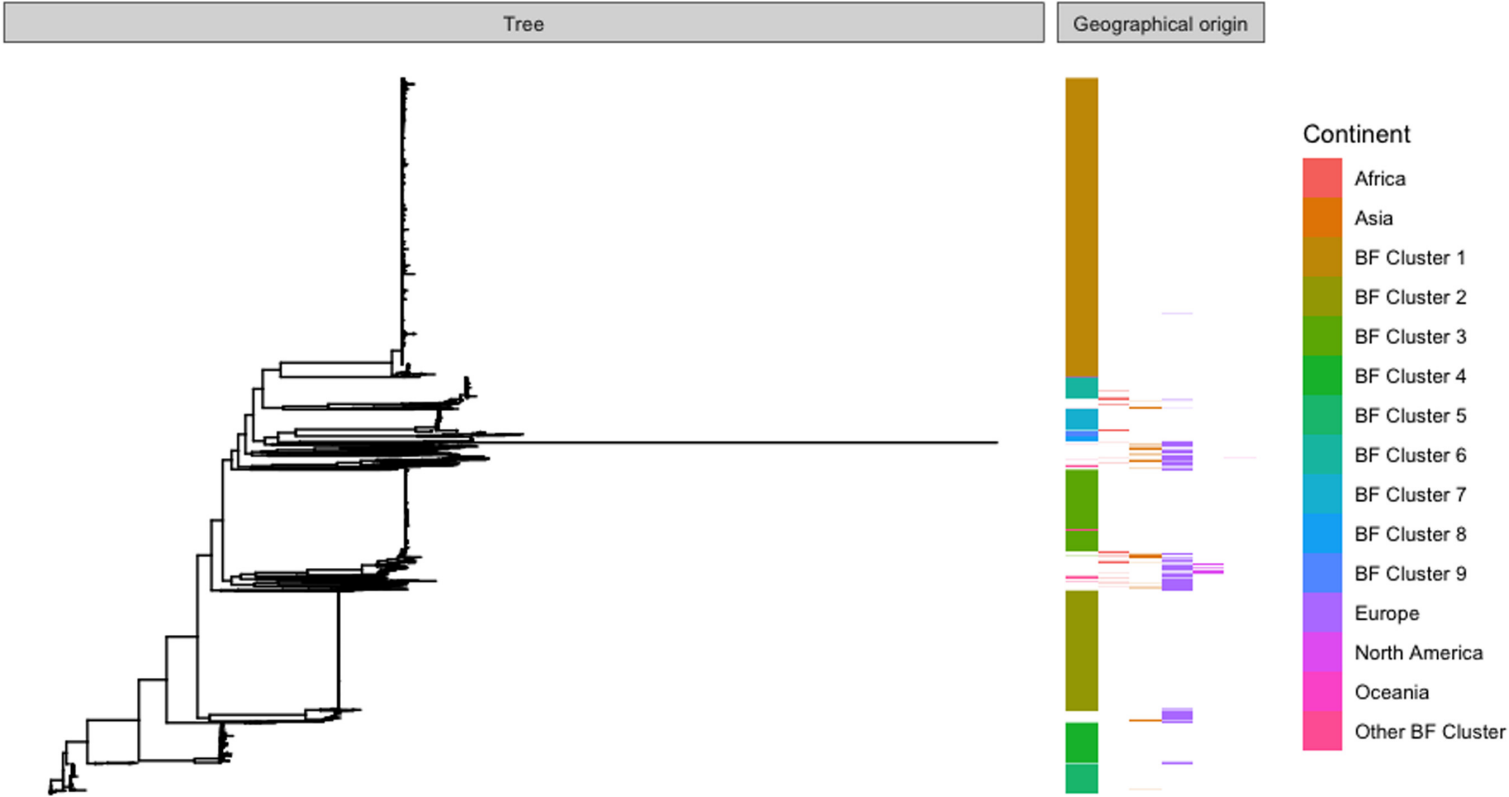
Core genome phylogeny of the Burkina Faso collection with the global background collection, arranged in order by first Burkina Faso and then alphabetical by continent, and annotated with colors for cluster, for Burkina Faso isolates, and continent of origin, for the global background collection (cf. Supplemental Table S4).

The PopPUNK clustering of the successfully sequenced Burkina Faso collection of 2838 carriage isolates returned 17 clusters, five of which were single isolate and three that had fewer than 10 isolates. These eight clusters were not considered in the downstream per-cluster analyses—the remaining nine clusters accounting for 99% of the isolate collection. These clusters broadly correspond to the dominant serogroups and sequence types (STs), as indicated in Figure 1: with cluster 1 being composed entirely of serogroup X and 96% of ST-181; cluster 2 composed 99.8% of serogroup Y and 98% of ST-4375; cluster 3 composed entirely of serogroup W and 98% of ST-11; cluster 4 composed 99% of serogroup W and 93% of ST-2881; cluster 5 composed 99% of serogroup Y and 59% of ST-767; cluster 6 composed entirely of serogroup A and 99% of ST-2859; cluster 7 composed wholly of nongroupable (NG) or capsule-null (cnl) isolates and 82% of ST-192; cluster 8 composed entirely of NG/cnl and 100% ST-198; and cluster 9 composed 100% of NG/cnl and 52% of ST-4899. Among these clusters, we found the major disease-causing lineages occurring in Burkina Faso pre- (clusters 3 and 6) and post-vaccine introduction (clusters 1 and 3) (Kristiansen et al. 2013). Disease surveillance since the implementation of the serogroup A vaccine in Burkina Faso has shown these lineages, clusters 1 and 3, to be the predominant causative strains of meningococcal IMD (Diallo et al. 2017). Based on the dominant serogroups and serogroup predictions for each cluster, we can see further that the clusters contain between 1% and 24% isolates which have likely varied their capsular phenotype. Figure 2 includes the entire Burkina Faso collection, as well as the global whole-genome sequences collected as described in the Methods. From the figure, we can see that the Burkina Faso population is composed of nine independently evolving lineages, where isolates from each cluster are more closely related to globally sampled isolates from their respective clusters as opposed to isolates sampled from other clusters within Burkina Faso.

### Cluster recombination rates

Average recombination event per mutation event (ρ/θ) rates for each cluster, as well as the overall average rate between all clusters, are shown in Table 1. These were calculated by averaging the per-branch ρ/θ rates inferred by Gubbins. In general, the rates were on the order of 10^−2^ with the average rate for the entire collection being 0.0745. The per-cluster average recombination rate ranged from 0.0082 to 0.1930, a difference of two orders of magnitude. The Kruskal–Wallis test on the per-branch per-mutation rates for each cluster returned an *H*-statistic of 440.977, with the associated *P*-value of 3.17 × 10^−90^, so there were at least two groups that were significantly different from one another. As the estimated average recombination rates are scaled to the different mutation rates of each cluster, the difference in recombination rates suggested that the clusters must be inherently different in terms of their recombination rate.

**Table 1.**
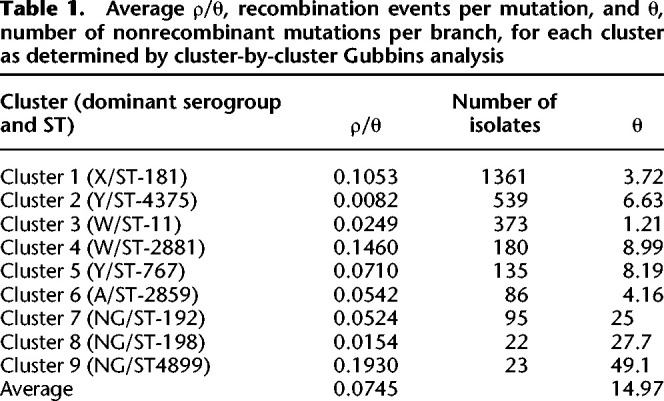
Average ρ/θ, recombination events per mutation, and θ, number of nonrecombinant mutations per branch, for each cluster as determined by cluster-by-cluster Gubbins analysis

**Figure 3.**
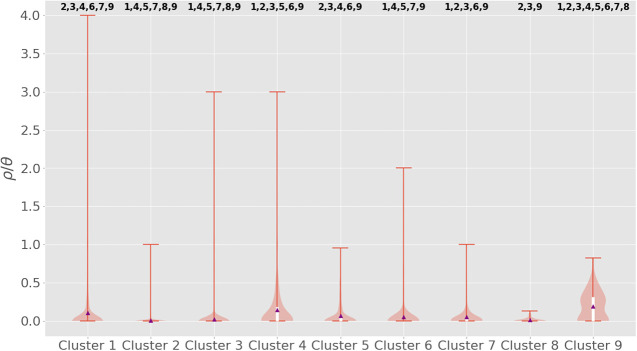
Violin plot of the per-isolate ρ/θ, recombination events per mutation event, as calculated by Gubbins for each cluster. The average ρ/θ per cluster is indicated by the purple triangles and these are also enumerated in Table 1. The *top* and *bottom* of the white boxes indicate the third and first quartiles, respectively, and the whiskers of the plot represent the maximum and minimum values. The orange background shading represents the distribution of inferred recombination rates within each cluster. Significant differences between clusters, as determined by a Kruskal–Wallis nonparametric analysis of variance on all the per-branch rates for each cluster, followed by post hoc statistical testing for differences between groups using Dunn's test and the conservative Holm-Bonferroni correction for multiple testing, is indicated by cluster numbers *above* each cluster's violin plot.

To determine which clusters were significantly different from one another, Dunn's test was used for post hoc statistical testing. The full results from this set of pairwise tests are shown in Figure 3 with markers for a significant difference from another cluster on the top of the individual violin plots. In general, the rates of recombination span three orders of magnitude, from 10^−1^ to 10^−3^, with clusters 1, 4, and 9 being on the order of 10^−1^, cluster 2 being on the order of 10^−3^, and the remainder being on the order of 10^−2^. This post hoc difference testing suggested that there are essentially three recombination phenotypes—the highly recombinant lineages (and serogroups): 1 (X/ST-181), 4 (W/ST-2881), and 9 (NG/ST4899), the moderately recombinant clusters/serogroups: 3(W/ST-11), 5(Y/ST-767), 6(A/ST-2859), 7(NG/ST-192), and 8(NG/ST-198), and the relatively nonrecombinant cluster 2 (Y/ST-4375).

### Within-cluster loci with elevated recombination rates

**Figure 4.**
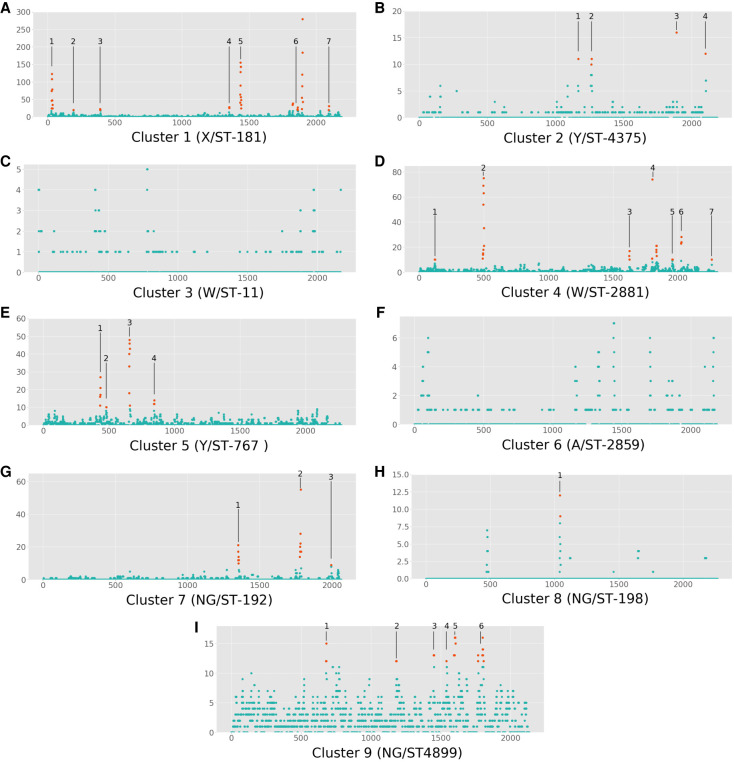
Per-cluster Manhattan plots of the number of recombinations per discrete 1000-base pair window in each cluster's unique reference genome. Plots for Clusters 1–9 are in panels *A*–*I*, respectively. The number of recombinations in a window is indicated on the *y*-axis, and the position on the genome, in kilobases, is on the *x*-axis. Regions with elevated rates of recombination, determined manually based on obvious peaks in the plot, are highlighted in orange, and the genes within each numbered region are fully described in Table 2.

Recombination across the genome for each cluster is summarized in Figure 4, and the genes contained within each cluster's candidate hotspots are fully described in Table 2. From these results, specifically those shown in Figure 4, we can see that in most clusters there are regions of the genome with substantially more recombination than the background level, though we cannot distinguish their relative levels of significance. Seven of the clusters had obvious peaks in the Manhattan plot of recombinations, whereas clusters 2 and 6 did not. Inspecting the annotation of the reference genomes for each cluster revealed a number of genes frequently present within these regions with elevated levels of recombination. In particular, *pilES*, the pilin genes, were present in clusters 1, 4, 5, 7, 8, and 9. Clusters 1, 4, 5, and 7 had regions containing genes associated with iron uptake. The transferrin-binding protein gene *tbpB* was present in clusters 1, 4, 5, and 7, and various portions of the bipartite outer membrane hemoglobin receptor gene *hpuA/B* were present in clusters 4 and 5.

**Table 2.**
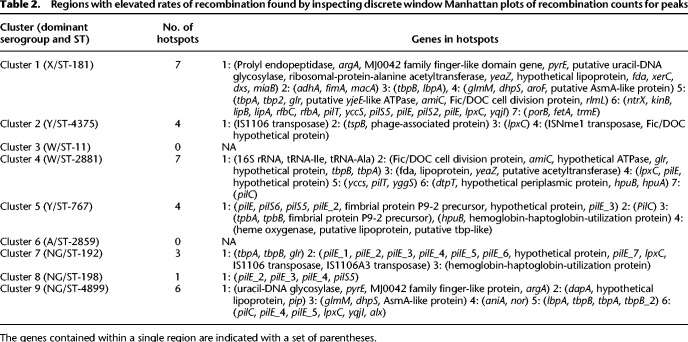
Regions with elevated rates of recombination found by inspecting discrete window Manhattan plots of recombination counts for peaks

### Recombination between clusters

**Figure 5.**
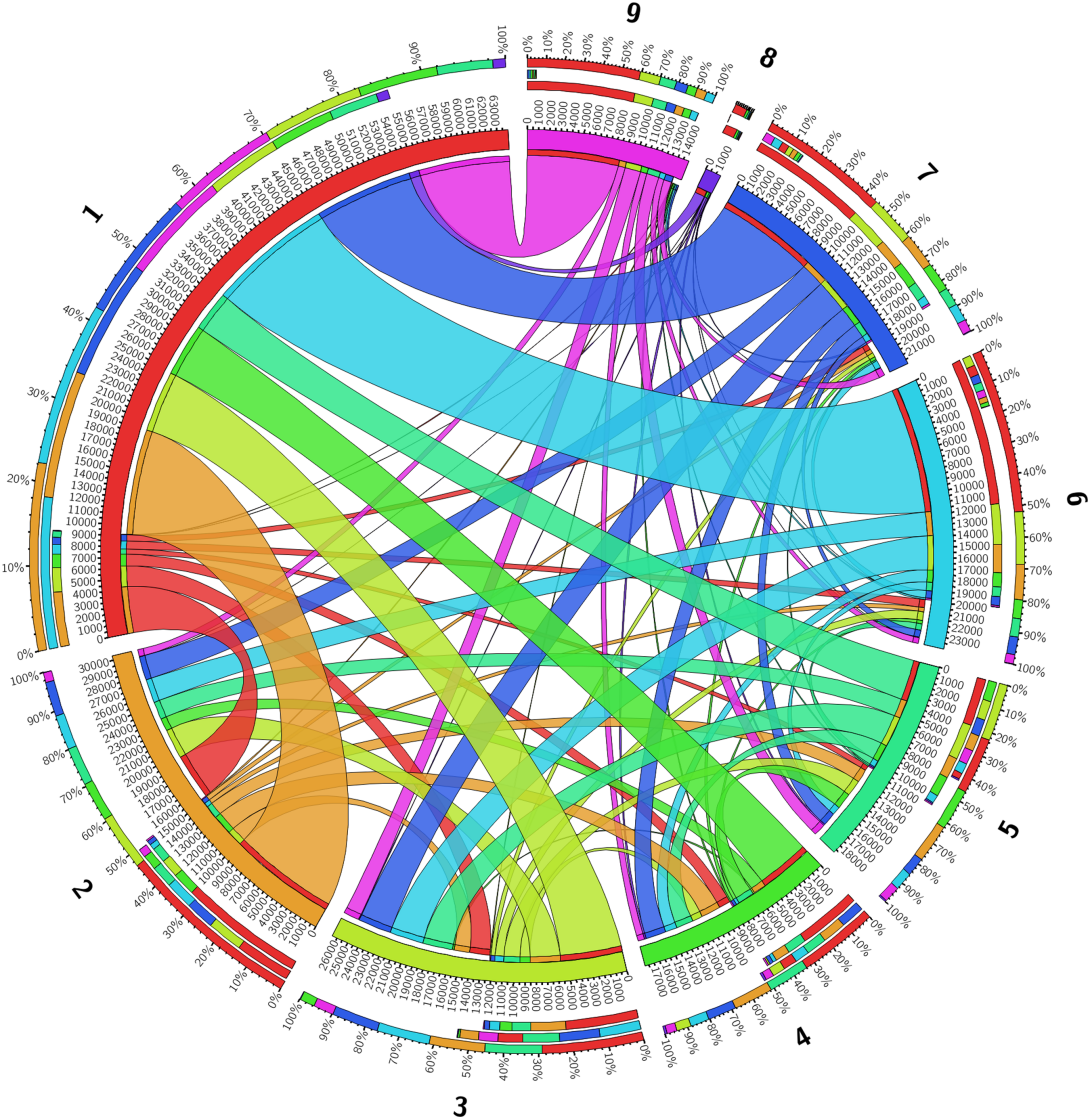
Chord diagram of the count of recombinant regions identified by fastGEAR between the clusters in the Burkina Faso *N. meningitidis* carriage collection. Clusters are positioned on the main circle of the diagram, with the arc length of the cluster indicating the number of recombination events. Linkages between clusters represent the number of recombinant regions occurring between those clusters with their width representing the number of regions and the color indicating the donating lineage. The three stacked bars *outside* the main diagram indicate, from *outermost* to *innermost*, the proportion of the total number of recombinant regions in each cluster colored by the other cluster involved, those same proportions only for the count of recombinant regions received, and those proportions for regions donated in the focal cluster.

The network of recombinant sequence region counts between donor and recipient clusters inferred from each gene in the pan-genome of the nine major clusters in the Burkina Faso collection is illustrated in Figure 5. A loose correlation between the size of each cluster and the number of contiguous recombinant sequences identified within was seen, though there were exceptions to this pattern that reflected the per-cluster estimates of recombination rate for each cluster. For instance, cluster 5 was involved in more exchanges of contiguous recombinant sequence than cluster 4, despite being smaller at 135 isolates compared to 280. This was also true of the smallest clusters 8 and 9, where 9 was involved in many more exchanges of contiguous recombinant sequence than 8.

Among the main lineages (clusters 1–9), clusters 1, 3, and 4 received more DNA by recombination than they donated, whereas 2, 5, 6, 7, 8, and 9 donated more DNA than they received. The results indicated that clusters with lower recombination rates, which make up a smaller proportion of the population (e.g., clusters 2, 5–9), donate more of the identified contiguous recombinant sequences than they receive, whereas clusters with a higher recombination rate or that are larger in population size, namely 1 and 4, and 1 and 3, respectively, receive more DNA than they donate.

Across the pan-genome of 2861 genes, the average number of contiguous recombinant sequences identified per gene was 63.03, with 1084 genes not having any recombinant sequences detected, and the number of recombinant sequences being deteced per gene ranging from 1 to 9015, in genes where a nonzero number of recombinant sequences were detected. Six genes had more than 5000 separate contiguous recombinant sequences inferred: an IS200-like transposase, the flavinyl transferase *apbE*, an unnamed P1 outer membrane protein, the microcin resistance gene *tldD*, the adhesin gene *mafA*, and the sRNA regulatory protein *yhbJ*.

### Selection and recombination in the pan-genome

*d*_N_/*d*_S_ was successfully estimated with SNPGenie for 1804 genes of the 2861 genes in the pan-genome, with the remaining genes being singletons or not having enough diversity to allow the calculation to be performed. The average *d*_N_/*d*_S_ for the pan-genome was 1.563, with values for individual genes ranging from 0 to 396.66. Relatively few genes, 119 of 1804, had a *d*_N_/*d*_S_ greater than one (Supplemental Table S2). Eleven genes had unusually high estimates for *d*_N_/*d*_S_—over 70—and the next highest *d*_N_/*d*_S_ estimate for a gene was 27. Six of these genes were hypothetical proteins without a known annotation, two genes were phage-associated and had some pseudogenized copies, and the last three genes were *hpallM*, a restriction enzyme, an uncharacterized peptidase, and the *exbD* membrane protein. As these genes are generally not well-described, they likely include artifacts from the annotation or pan-genome inference stage or are simply unusual regions of the genome that do not evolve like other genes, resulting in unusually high *d*_N_/*d*_S_ estimates for these genes. Consequently, these 11 genes were excluded from figures and further analysis.

**Figure 6.**
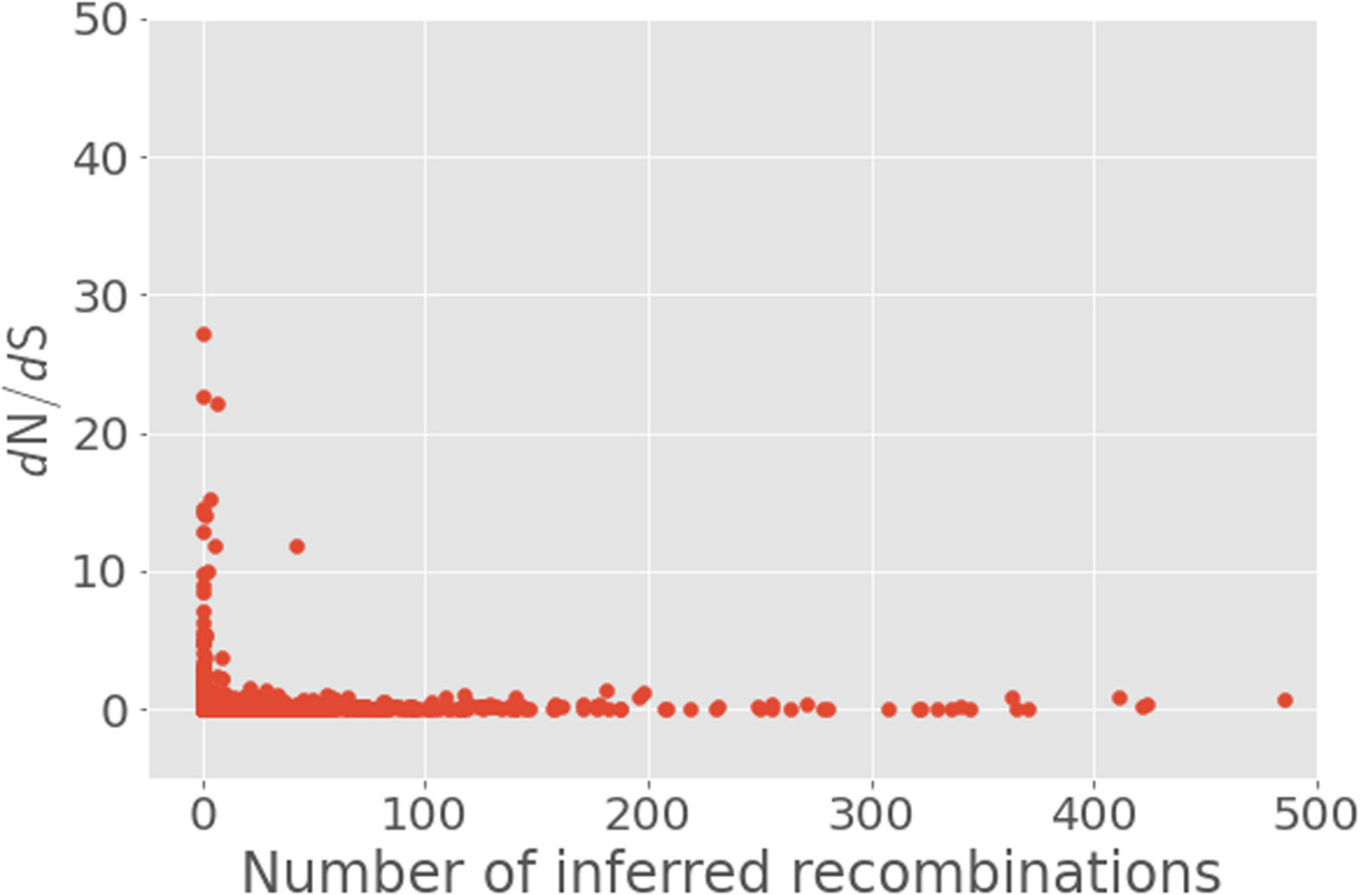
Scatter plot of number of recombination events versus *d*_N_/*d*_S_ for genes in the pan-genome of the entire Burkina Faso collection of carriage isolates. Genes with abnormally high estimates of *d*_N_/*d*_S_ were excluded from the plot as described in the text.

Even with those genes excluded, correlating the *d*_N_/*d*_S_ and number of separate recombinant sequence regions identified per gene in the pan-genome with a nonparametric Spearman's rank correlation returned a highly significant negative correlation coefficient of −0.122, with *P* = 2.21 × 10^−6^. As can be seen in Figure 6, however, one gene has a *d*_N_/*d*_S_ value of over 10 and 42 separate recombinant sequence regions identified within. This is *dpnA*, a DNA methylation modification enzyme. Additional analysis of this gene with BUSTED and FUBAR confirmed that it is under gene-wide selection (BUSTED, *P* = 0.00015) and that a single amino acid site is under selection (FUBAR, posterior probability = 0.9039). The 42 branches of the phylogeny which featured the recombination were also tested with aBSREL to see whether they were under selection, and one of these branches was found to be under selection (*P* = 0.00059).

## Discussion

The first, immediate conclusion from the composition of our sample of *N. meningitidis* carriage isolates from Burkina Faso is that, over the course of the sampling period, the population was composed primarily of nine independently evolving lineages. Bacteria of these lineages were more closely related to isolates of the same lineage from other parts of the world than to isolates from other lineages within Burkina Faso, as indicated by Figure 2, reflecting the deeply stratified population structure of *N. meningitidis*, where clonal lineages with a distant common ancestor co-exist in space and time, evolving largely independently. Seven of the lineages making up the Burkina Faso population were present in all four years of sampling. Two lineages, however, were not consistently detected. Cluster 3, the serogroup W/ST-11 lineage, was first detected in our collection in 2011 and then also in 2012 but was not present in 2009 or 2010, although it caused a large epidemic in Burkina Faso in 2002 (Koumaré et al. 2007). This may therefore represent either a re-introduction of the lineage after it had gone locally extinct or an increase in population size to be once again detectable after the population perturbation caused by vaccination. Cluster 6, the serogroup A lineage, is the other cluster which was not detected throughout our sampling period, being found in 2009, 2010, and a single isolate in 2012. The isolate in 2012 was substantially divergent from the rest of the lineage (Supplemental Fig. S1) and belonged to a different sequence type, ST-7, whereas the earlier isolates of the lineage belonged to ST-2859. Given that this lineage was the target of the vaccine introduced in Burkina Faso at the end of 2010, it seems likely that the original serogroup A lineage was extinct by 2011 in Burkina Faso and the 2012 isolate represents a re-introduction from abroad. The dynamic changes in the composition of our population underscore the need for carriage surveillance in monitoring the evolution of *N. meningitidis* in order to guide future intervention. Though there were shifts in the population composition of our sample, including lineages which were restricted to a subset of the sampling period, a majority of the lineages that made up the study population were present throughout and therefore do reflect a fluctuating yet consistent population in Burkina Faso across the time period of our sampling.

The significant differences in the levels of recombination between different clusters is an important and interesting result. As far as we are aware, this is the first study of a large, contemporaneous population of *N. meningitidis* that has used whole-genome data to estimate and compare the recombination rates of different lineages within the population. Though it has been shown in other species and with more disparate sampling, we show here that, even within narrow geographical and temporal constraints, the lineages composing this population of *N. meningitidis* differ in their propensity to recombine by orders of magnitude. This has important implications for how likely it is for any bacterium from a given lineage to pick up genetic material from another lineage, potentially a disease-causing one. This is of particular importance with regard to the efficacy of current vaccines and the design of future ones.

The rate of recombination within clusters was not entirely determined by the clusters’ relative size in the population—and hence stochastic opportunity, assuming that the proportions of lineages in our sample reflect those in the general population—and though recombination rate is doubtlessly affected by many factors, this does suggest that there are lineage-specific genetic components affecting the rate of recombination in *N. meningitidis*. Though there exists an understanding of the mechanisms that underlie recombination in the species (Obergfell and Seifert 2015), knowledge of how these and other genetic factors that affect the rate of recombination differ between lineages has not yet been fully explored. This awareness would not only be important to our understanding of the evolution of *N. meningitidis* and other *Neisseria*, but it would also allow for a more detailed monitoring of the populations of these serious human pathogens. Finally, it is worth noting that the highest rate of recombination, more than twice the average, was seen in one of the NG clusters, cluster 9. Nonserogroupable *N. meningitidis*, which may lack the whole capsule operon or simply have loss-of-function mutations in key capsular genes, are rarely implicated in disease. However, given the extremely high recombination rate of the nongroupable cluster 9, some NG/cnl *N. meningitidis* bacteria may play a larger role in the evolution of a geographical population than their frequency would suggest. Therefore, it may prove worthwhile to monitor NG/cnl *N. meningitidis* more closely in future study.

Recombination in the pan-genome of all the lineages follows a pattern that is loosely consistent with the estimated within-cluster rates. Clusters with higher estimates of average recombination rate, ρ/θ, also generally have more inferred within-gene separate contiguous recombinant sequence in the pan-genome than clusters with lower estimates of ρ/θ, when the population size of the cluster is taken into account. For instance, clusters 8 and 9 have a similar population size but differ greatly in the number of between-cluster recombinant sequences detected in each cluster. It is clear, though, that the primary factor that determines how much recombination lineages undergo in the pan-genome is the proportion of each cluster within the population at large, and hence the stochastic opportunity of recombining, particularly in terms of the likelihood of an encounter between two random individuals of different lineages. This is in contrast to the within-lineage recombination rates which were not correlated with population size. The recombination pattern in some clusters also suggests that the inherent recombination rate of a lineage affects how recombination proceeds between itself and other lineages. For example, cluster 2, despite being the second largest cluster, donates DNA more often than it receives DNA, like the much smaller clusters 5–9. This is as one would predict, as the donation of DNA in a recombination event is governed principally by the rate at which bacteria from different lineages physically encounter one another in the same host, whereas receiving DNA is further affected by how likely the receiving bacteria is able to take up the DNA. Therefore, in lineages with low inherent recombination rates, such as cluster 2, we expect to see a bias toward donating rather than receiving of DNA, compared to what is expected based on the size of the cluster. Though this effect is clearly dwarfed by the effect of cluster size in general, this suggests that more recombinant lineages of *N. meningitidis*, may be able to incorporate and maintain more variation from other lineages, including from lineages that themselves are not particularly recombinant (Supplemental Fig. S2). Although this may not be particularly surprising, it suggests that the inherent recombination rate has important consequences for how flexible the genome of a lineage is likely to be in response to different selection pressures and how likely it is that it may become more virulent, antibiotic-resistant, or escape a vaccine.

The radically different amounts of recombination in different regions across the genome of most lineages within this population, combined with the discovery of consistent genes within regions of elevated recombination, suggest two things: first, that recombination in the *N. meningitidis* genome is elevated in certain regions; and second, that multiple lineages within the population are experiencing routine ongoing adaptation to human hosts. In particular, the elevated rates of recombination in pilin and iron uptake genes—found across clusters 1, 2, 4, 5, 7, 8, and 9, as well as in previous work (Linz et al. 2000)—corroborates that these genes are crucial determinants of the ability of *N. meningitidis* to survive within a human host. Pilin genes, expressed on the surface of the bacteria, are targeted by the immune system (Wachter and Hill 2016), and the *pilE* gene is further known to be highly recombinant (Aho et al. 2005), due in part to the various mechanisms of phase variation. Human hosts are typically very iron-deficient environments for bacteria to grow in, resulting in a strong selective pressure on iron uptake pathways (Perkins-Balding et al. 2004; Yu et al. 2014), and iron-binding proteins have been shown to be very diverse and have repeats which may promote both intra- and inter-genomic recombination close by (Acevedo et al. 2014). Further work should explore methods of determining which of these regions of elevated recombination are statistically significant and consistently so across data sets which are more temporally and geographically diverse, to fully explore the genetic and evolutionary factors driving elevated recombination rates in these regions.

The pattern of recombination events in the per-gene analysis of the pan-genome is largely as expected given the within-cluster results, with an IS200 transposase, surface-exposed genes such as the adhesin *mafA*, and the microcin resistance gene *tldD* among the top five most recombinant genes. Other genes expected to be highly recombinant, such as the iron uptake genes *tbp2*/*tpbB* and the surface-exposed T cell stimulating protein *tspB*, also have many inferred recombinations and are among the top 15 most recombinant genes. Although these genes are known to be generally very recombinant in *Neisseria*, it is also likely that the disruption of the population by vaccination has caused substantial shifts in the population size of each lineage which, in most cases, apart from cluster 6, was likely a population expansion. During this demographic shift, lineages will have had more opportunity to selectively adapt to different hosts and also persist to exchange DNA, and these events are presumably contributing to the consistently high levels of recombination detected in different genes, particularly immune targets, across the different lineages.

The *d*_N_/*d*_S_ values for each gene in the pan-genome follow a pattern that is broadly expected, with most values being very low, and relatively few genes—119—having a *d*_N_/*d*_S_ > 1. It is difficult to draw specific conclusions from a simple gene-wide estimate of *d*_N_/*d*_S_, but the significant negative correlation with the number of separate contiguous recombinant sequences for each gene in the pan-genome and the gene's estimated *d*_N_/*d*_S_ can inform us as to the general evolutionary role played by recombination in this population. One of the evolutionary explanations of the maintenance of recombination in populations has been that it prevents the accumulation of deleterious mutations (Felsenstein 1974). Given the significant negative correlation count of recombinant sequences and *d*_N_/*d*_S_ in the pan-genome, and the fact that nonsynonymous mutations are generally more likely to be deleterious, avoiding the accumulation of deleterious mutations, or enhancing the effect of negative selection, seems to be the primary role played by recombination in this population. In spite of this, a single gene in the pan genome, the *dpnA* DNA methylation modification enzyme, has both a substantial number of recombinant sequences, 42, and an elevated *d*_N_/*d*_S_ estimate of 11.8. All of these sequence regions identified as recombinant originate from an unknown bacterium outside the collection, and 41 ended up in the cluster 1 lineage, with the final one ending up in cluster 2. Further analysis of this gene with the BUSTED algorithm for detecting gene-wide episodes of selection confirmed that it has been under selection, and using the FUBAR method for detecting specific sites under selection confirmed that the *dpnA* gene has definitely been under selection in this population. Though the specific site under selection detected by FUBAR did not overlap with any of the sequence regions identified as recombinant, testing the branches of the gene phylogeny with the recombination present with aBSREL found one of the lineages possessing the recombinant sequence to be under selection as well. This example of a high recombination gene under selection is an exception to the general pattern of recombination in the Burkina Faso population of *N. meningitidis* and suggests that recombination may be acting here to speed adaptation instead of simply preventing the accumulation of deleterious mutations. Though recombination acting in this manner has long been theorized to be true for all recombining organisms under the Fisher-Muller model of recombination, and shown experimentally with plasmids in *Escherichia coli* (Cooper 2007), we demonstrate this phenomenon here in a sample from a natural population.

Evidence of adaptation speeded by recombination was seen in the *dpnA* gene in the cluster 1 lineage. Though it is uncharacterized in *N. meningitidis*, the *dpnA* gene, as part of the DpnII restriction-modification system, has been studied in *Streptococcus pneumoniae*, another bacterial pathogen that primarily colonizes the nasopharynx of healthy humans. In *S. pneumoniae*, *dpnA* is specifically produced during natural competence for promoting recombination by methylating incoming DNA to protect it from the rest of the DpnII system (Johnston et al. 2013). Though we cannot assume that *dpnA* has an identical function in *N. meningitidis*, given the important consequences that DNA methylation is known to have for recombination in *N. meningitidis* (Seib et al. 2017; Kim et al. 2019) and the on-average higher rate of recombination in cluster 1 isolates with this *dpnA* recombination (0.159 vs. 0.105), it seems that *dpnA* recombination may have affected the overall recombination phenotype in the cluster 1 lineage.

The effects of recombination on the evolution of various genes in the *N. meningitidis* genome have been the subject of extensive research. This research has largely been focused on the effects of recombination in a specific set of genes and over a long period of time (Vigué and Eyre-Walker 2019). However, our understanding cannot be complete without also investigating how recombination affects the evolution of a population of *N. meningitidis* in the short to medium term. Without this, it would be difficult to predict how different populations will respond to vaccines, anticipate changes in virulence between different lineages, or know if a population has a high risk of developing antimicrobial resistance. In this study, we have conducted such an analysis on a collection of *N. meningitidis* carriage samples from Burkina Faso. We have demonstrated how using high-throughput whole-genome sequencing and subsequent computational analyses on whole-genome data allow for the possibility of understanding the differential effects of recombination on the evolution of the various lineages within this population, how the lineages of the population are interacting with each other, and the evolutionary explanations which underlie these patterns.

## Methods

### Sample collection and laboratory analysis

To assess the impact of the monovalent serogroup A conjugate vaccine on meningococcal carriage, 50,811 samples were collected over the course of four years, from 2009 to 2012, in 10 rounds of sampling. This took place across three sites in Burkina Faso: the Bogodogo arrondissement of the capital city, Ouagadougou; 10 villages in the Kaya district, 100 km northeast of Ouagadougou; and 10 villages in the Dandé district, 350 km west of Ouagadougou. Oropharyngeal swabs were taken from healthy volunteers, and associated metadata were collected from the individuals. A total of 2848 meningococcal isolates were recovered from the 10 samplings (overall carriage rate 6.05%) and confirmed as *N. meningitidis* at the Norwegian Institute of Public Health (NIPH), Oslo (Kristiansen et al. 2011, 2013, 2014). The isolates were serogrouped using commercial antisera (Remel), most of them were characterized by multilocus sequence typing (Maiden et al. 1998), and all were stored frozen in Greaves medium (Craven et al. 1978) at −70°C.

### Whole-genome sequencing, quality control, assembly, and annotation

DNA was extracted from 2839 of the 2848 collected isolates and these were sent to be sequenced at the Wellcome Sanger Institute, using Illumina HiSeq 2000 sequencing technology. Paired-end libraries were prepared with insert sizes of 500 base pairs, for 125-base pair reads, sequenced at a read depth of 100×. The quality of the raw sequencing reads was assessed using an internal quality control (QC) pipeline as well as a local implementation of Kraken (Wood and Salzberg 2014), the metagenomic sequence classifier, to check for contamination. Raw reads from 2838 isolates passed this QC and were then used to produce de novo genome assemblies (Supplemental Table S3) using the bacterial assembly pipeline at the Sanger Institute (Page et al. 2016). The resulting genome assemblies were annotated using Prokka (Seemann 2014), the prokaryotic annotation pipeline, to infer gene content. To identify the ST of all isolates from the sequence data, we used SRST2 (Inouye et al. 2014), the *Neisseria* PubMLST sequence typing database, and the raw reads for each isolate. Finally, in order to determine serogroups for each isolate in silico, we adapted seroBA (Epping et al. 2018) using the published capsule reference sequences (Harrison et al. 2013). In addition to the collected isolates from Burkina Faso, to give global genetic context, genomes of an additional 427 publicly available *N. meningitidis* isolates from five continents and maximizing serogroup diversity, as well as the FA1090 *Neisseria gonorrhoeae* reference as an outgroup, were downloaded from the European Nucleotide Archive (ENA; https://www.ebi.ac.uk/ena/browser/home) and processed using the same quality control, assembly, annotation pipeline, and further analyses as above where applicable (Supplemental Table S4).

Selected isolates from five of the clusters in the collection were resequenced with Oxford Nanopore MinION at the NIPH, with R9.4 (FLO-MIN106) flowcells and the SQK-RBK001 rapid barcoding kit for 1D reads, in order to produce reference genomes for those clusters. De-multiplexing and base-calling was performed using Albacore version 2.1.2 (Sahoo 2017), and then porechop version 0.2.3 (Wick et al. 2017a) was used to remove chimeric reads and adapters from the nanopore reads. The reference genomes were then assembled by hybrid assembly using both the original Illumina sequence and the nanopore reads, with Unicycler (Wick et al. 2017b) before being annotated with Prokka (Seemann 2014) like the rest of the sequenced isolates.

### Clustering and phylogeny inference

To assess the population structure of the collection of carrier isolates, we clustered the sequence assemblies using PopPUNK (Lees et al. 2019). For four clusters among the nine largest carried forward for further analysis, an Oxford Nanopore-sequenced reference was not available. Therefore, the assembly with the lowest number of contigs was selected for further scaffolding, using the MeDuSa multidraft assembly scaffolder (Bosi et al. 2015) and the rest of the isolate assemblies from that cluster. The resulting scaffolds for each cluster were then joined with gaps of 1000 blank nucleotides. The resulting sequences, or the Oxford Nanopore-sequenced references, were then used as reference genomes in producing whole-genome pseudoalignments for each cluster. We did this using a custom mapping, variant calling, and local realignment around indels pipeline using BWA-MEM (Li 2013), SAMtools mpileup (Li 2011), and MUSCLE (Edgar 2004), and then used the resulting whole-genome pseudoalignments to infer phylogenies for each cluster, using Gubbins (Croucher et al. 2015), and RAxML, its underlying dependency (Stamatakis 2014).

In order to construct a phylogeny for the entire collection and also the collection combined with global isolates, we used the recently published Panaroo pan-genome pipeline (Tonkin-Hill et al. 2020) to infer a set of core genes for the entire collection. We concatenated alignments of all the core genes and then used IQ-TREE (Nguyen et al. 2015), with the substitution model, GTR + F+I + G4, inferred by ModelFinder (Kalyaanamoorthy et al. 2017), to infer phylogenies for both the entire Burkina Faso collection and the Burkina Faso collection plus global isolates.

### Recombination, selection, and pan-genome analyses

Recombination was first analyzed cluster-by-cluster, using whole-genome pseudoalignments of each cluster, generated as described above, and Gubbins (Croucher et al. 2015). Gubbins outputs an estimate of ρ/θ, the number of recombination events per mutation event, for each branch of the phylogeny (Supplemental Table S1). To test if any of the differences in recombination rates between clusters were statistically significant, we used a Kruskal–Wallis nonparametric analysis of variance (Kruskal and Wallis 1952) on all of the estimated per-branch rates for each cluster, followed by Dunn's test for post hoc statistical testing (Dunn 1964) for differences between groups and the conservative Holm-Bonferroni correction for multiple testing (Holm 1979). Candidate recombination hotspot regions in the reference genome of each cluster were found by producing Manhattan plots of the number of recombination events per 1000-base pair discrete window across the genome, manually looking for peaks in these plots, and comparing them to a Prokka annotation of the genome to establish which genes, if any, are within these windows.

To then analyze the recombinations between clusters in the entire Burkina Faso collection, we ran fastGEAR (Mostowy et al. 2017) on alignments of each gene of the Burkina Faso collection's pan-genome, as inferred by Panaroo (Tonkin-Hill et al. 2020) and aligned using MAFFT (Katoh and Standley 2013), using the whole-genome clustering inferred by PopPUNK (Lees et al. 2019). fastGEAR proceeds base pair-by-base pair in a multiple sequence alignment to identify recombinant regions by identifying the most likely lineage of origin for each base pair and, as such, identifies contiguous sequence regions whose origin is more likely to be from a different lineage that a given isolate's own and hence is recombinant. Although this approach does lead to some uncertainty with regard to how the number of these regions correspond to the number of physical events, this is not a confounding bias. The further advantage of fastGEAR as a method for detecting recombination is in how it infers recombination explicitly with directionality, allowing the flow of recombination events between clusters to be visualized as a network. This was done using Circos Table Viewer and Circos (Krzywinski et al. 2009). To assess the effect of selection on the pan-genome, *d*_N_/*d*_S_ ratios were also inferred for each gene using the same alignments as input into fastGEAR and the implementation of the Nei-Gojobori method (Nei and Gojobori 1986) of calculating *d*_N_/*d*_S_ as implemented in SNPGenie (Nelson et al. 2015). These *d*_N_/*d*_S_ results were then correlated with the number of recombination events per gene using a nonparametric Spearman's rank correlation (Spearman 1904). Genes with an elevated SNPGenie *d*_N_/*d*_S_ estimate and a high number of recombinations were further analyzed with the HyPhy package (Pond and Muse 2005), in particular, the BUSTED (Murrell et al. 2015) method for detecting gene-wide episodic selection, the FUBAR (Murrell et al. 2013) method for finding codons under selection, and the aBSREL (Smith et al. 2015) method to find selected branches in a phylogeny.

The SciPy (Virtanen et al. 2020), pandas (McKinney 2010), and Matplotlib v. 2.2.3 (Hunter 2007) Python libraries were used throughout this study for statistics, data manipulation, and data visualization, respectively. The R (R Core Team 2019) package ggtree (Yu et al. 2017) was used to draw phylogenies.

## Data access

All raw sequencing data generated in this study have been submitted to the European Nucleotide Archive (ENA; https://www.ebi.ac.uk/ena) under the study accession number PRJEB12668.

Metadata, assemblies, and sequence types for all the isolates in the Burkina Faso collection are available on *Neisseria* PubMLST (https://pubmlst.org/neisseria/), as per Kristiansen et al. (2013). Whole-genome sequenced isolates are tagged with their ENA run accessions. Interactive notebooks for recreating analyses are included as Supplemental Code.

## Supplementary Material

Supplemental Material
