# Supplementary material for: The effect of recombination on the evolution of a population of Neisseria meningitidis
Source: Genome Res. 2021 Jul;31(7):1258–68. doi: 10.1101/gr.264465.120 (PMC8256868; doi:10.1101/gr.264465.120)
Supplement: Supplemental Material [file supp_31_7_1258__DC1.html]

The effect of recombination on the evolution of a population of Neisseria meningitidis — Supplemental Material 

# The effect of recombination on the evolution of a population of *Neisseria meningitidis*

## Supplemental Material

- Supplemental\_Fig\_S1.pdf
- Supplemental\_Fig\_S2.pdf
- Supplemental\_Table\_S1.csv
- Supplemental\_Table\_S2.csv
- Supplemental\_Table\_S3.csv
- Supplemental\_Table\_S4.txt
- Supplementary\_Code\_S1.zip
