# Supplementary material for: The effect of recombination on the evolution of a population of Neisseria meningitidis
Source: Genome Res. 2021 Jul;31(7):1258–68. doi: 10.1101/gr.264465.120 (PMC8256868; doi:10.1101/gr.264465.120)
Supplement: Supplemental Material [file supp_gr.264465.120_Supplemental_Fig_S1.pdf]

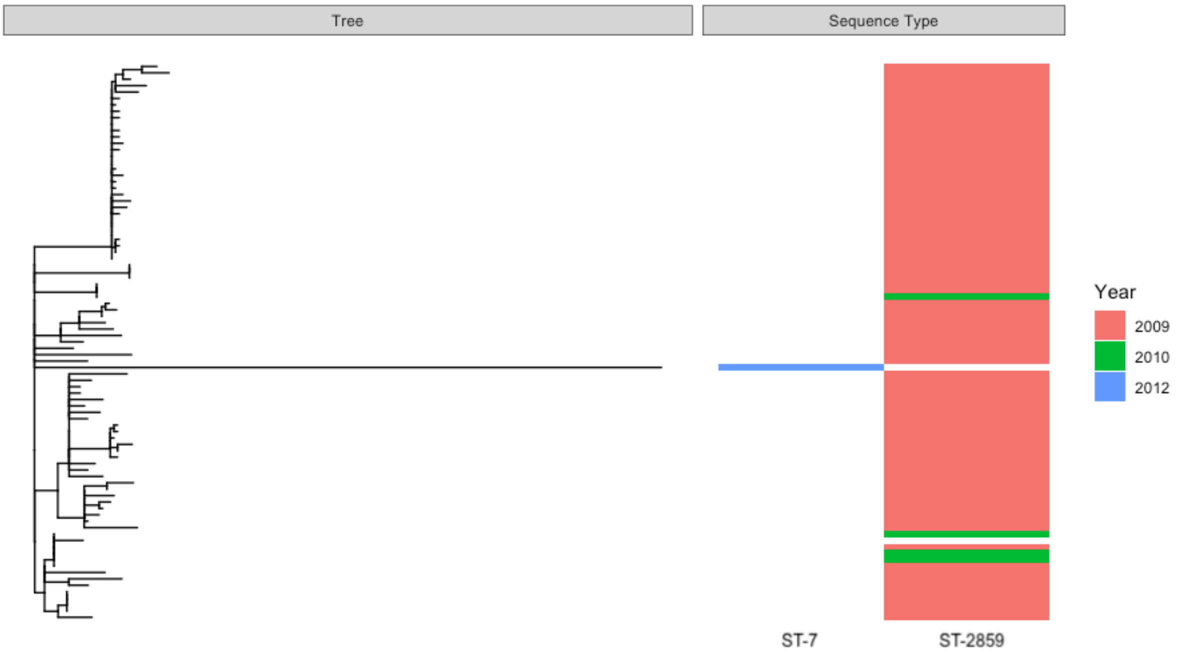

Supplementary Figure 1: Whole genome phylogeny of the PopPUNK cluster 6, serogroup A lineage, annotated with sequence types and years of isolation. Sequence types are indicated by the column, and year of isolation by the colours as per the legend.
