# Supplementary material for: The effect of recombination on the evolution of a population of Neisseria meningitidis
Source: Genome Res. 2021 Jul;31(7):1258–68. doi: 10.1101/gr.264465.120 (PMC8256868; doi:10.1101/gr.264465.120)
Supplement: Supplemental Material [file supp_gr.264465.120_Supplemental_Fig_S2.pdf]

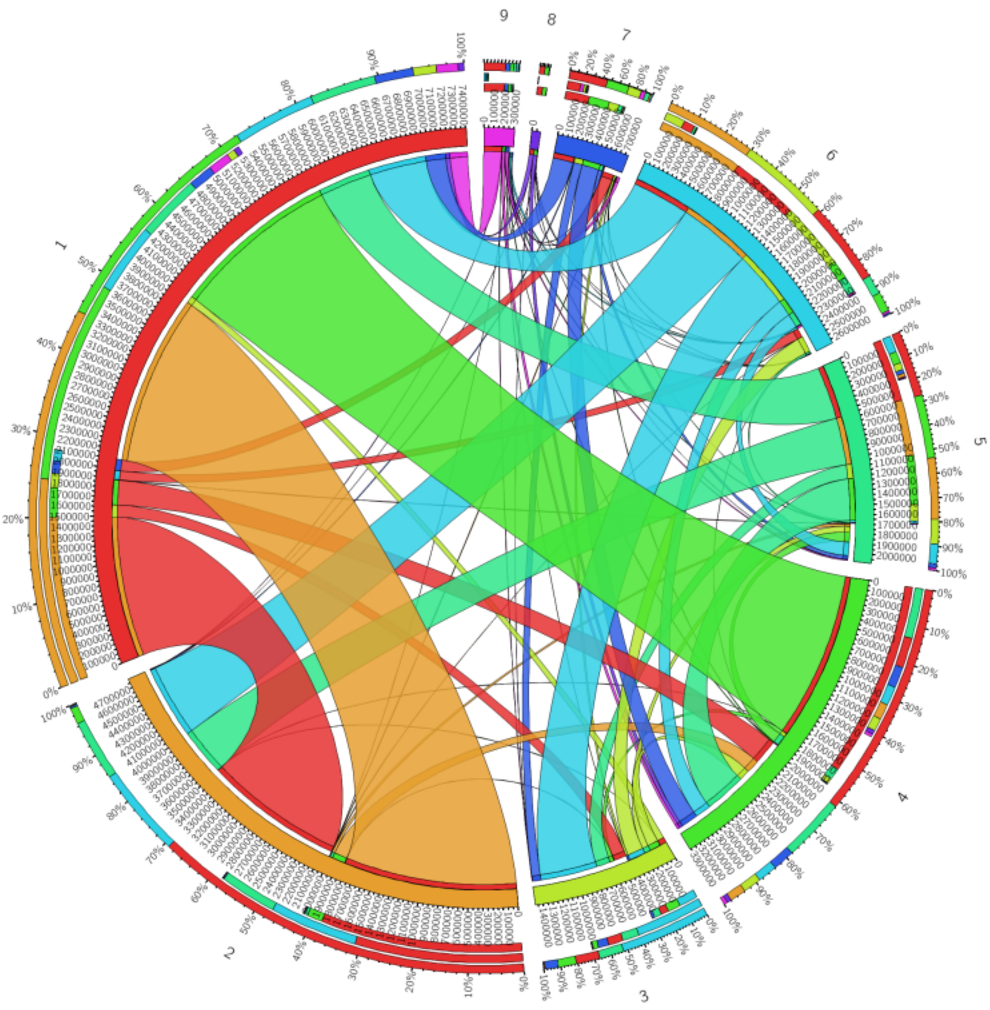

Supplementary Figure 2: Chord diagram of the number of base pairs in regions identified as recombinant by fastGEAR between the clusters in the Burkina Faso *N. meningitidis* carriage collection. Clusters are positioned on the main circle of the diagram, with the radial length of the cluster indicating the number of recombinant base pairs. Linkages between clusters represent the number of recombinant base pairs occurring between those clusters with their width representing the number of recombinant base pairs and the colour indicating the donating lineage. The three stacked bars outside the main diagram indicate, from outermost to innermost, the proportion of the total number of recombination events in each cluster coloured by the other cluster involved, those same proportions only for base pairs received, and those proportions for base pairs donated for the focal cluster. To validate the conclusions reached by examining the counts of recombinant regions identified by fastGEAR, and their origin and destination clusters, we examine the same relationship, but instead for the total number of base pairs identified as recombinant, in effect accounting for the lengths of the recombinant regions identified. As we can see from this figure, the general trend of larger clusters being more recombinant than smaller clusters holds, as do the exceptions to that trend based on recombination rate. Cluster 1 is the most recombinant, but cluster 9 is substantially more recombinant than 8, and 6 more recombinant than 5, despite the former clusters being smaller in both cases.
